# Polarity‐Controlled Coexistence of Electrochemical Metallization and Valence Change Mechanisms in a Unified Metal Halide Perovskite Memristor

**DOI:** 10.1002/advs.78211

**Published:** 2026-10-26

**Authors:** Ismael Fernandez‐Guillen, Omar E. Solis, Pablo F. Betancur, Rafael Abargues, Pablo P. Boix

**Affiliations:** ^1^ Institute of Materials Science of the University of Valencia Paterna, València Spain; ^2^ CNRS Centre de Recherche sur l'Hétéro‐Epitaxie et ses Applications (CRHEA) Université Côte d'Azur Valbonne France; ^3^ Instituto de Tecnología Química (Consejo Superior de Investigaciones Científicas – Universitat Politècnica de València) Valencia Spain

**Keywords:** ECM, electroforming, memristor, metal halide perovskite, performance, polarity, VCM

## Abstract

Metal halide perovskites (MHPs), owing to their mixed ionic‐electronic conductivity and defect‐tolerant lattice, have emerged as promising candidates for next‐generation memristors. Yet, the interplay of distinct resistive switching mechanisms remains poorly understood. Here, we provide direct experimental evidence that two distinct resistive switching mechanisms, electrochemical metallization (ECM), driven by the reactive metal electrode, and the valence change mechanism (VCM), mediated by intrinsic ion migration within the perovskite lattice, can coexist within the same perovskite memristor and can be selectively activated by simply reversing the electroforming voltage polarity, without altering the device structure or active layer composition. This controllable switching enables a systematic comparison of their intrinsic performance. ECM‐driven devices exhibit low operating voltages (+0.18/−0.3 V), high endurance (> 10^4^ cycles), and moderate retention (∼ 4 × 10^3^ s), whereas VCM switching achieves superior data retention (∼ 7 × 10^3^ s) and a higher resistance contrast (∼ 10^5^), albeit with higher operating voltages and reduced cycling stability. Replacing the active Ag electrode with inert contacts confirms the absence of ECM and isolates intrinsic ion migration as the origin of VCM switching. These findings establish a clear framework for distinguishing and engineering operating mechanisms in MHP‐based memristors, providing design guidelines for reliable, multifunctional memory and neuromorphic devices.

## Graphical abstract

Electrochemical metallization and valence change mechanisms are shown to coexist within a single metal halide perovskite memristor, selectively activated by simply reversing the electroforming voltage polarity. Ag‐driven switching delivers low operating voltages and high endurance, while intrinsic ion migration yields superior retention and resistance contrast. This polarity‐controlled duality establishes a practical framework for disentangling switching mechanisms and engineering next‐generation perovskite‐based memory and neuromorphic devices.

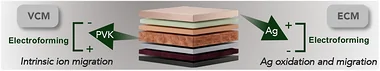

## 1 Introduction

The escalating demand for data processing and the corresponding rise in energy consumption emphasize the urgent need to develop innovative technologies based on novel materials capable of overcoming persistent computing‐related challenges such as the von Neumann bottleneck and the scaling limits imposed by Moore's law [1, 2].

Among these next‐generation technologies, memristors, which encompass a class of non‐volatile resistive switching (RS) memory devices [3, 4], represent a disruptive alternative to conventional charge‐based memories such as flash technologies [5]. Unlike traditional memory, memristors encode information by reversibly switching between a high‐resistance state (HRS) and a low‐resistance state (LRS) in response to electrical stimuli, allowing them to retain data even in the absence of power. This non‐volatility, combined with low power consumption, high resistance contrast, and nanoscale scalability, makes memristors highly attractive for future memory and logic architectures [6, 7, 8]. Beyond high‐density resistive random‐access memory (RRAM), tunable conductivity enables multiple intermediate states, making them highly promising for neuromorphic computing with analog and synaptic‐like behavior [9, 10, 11].

Within the landscape of advanced materials, metal halide perovskites (MHPs) offer a unique advantage due to their rich set of optoelectronic and ionic properties, as well as their compatibility with low‐cost, solution‐based fabrication techniques [12, 13, 14, 15]. MHPs are semiconductors with the general formula ABX_3_, where A is a monovalent cation (e.g., MA^+^, FA^+^, Cs^+^), B is a divalent metal (typically Pb^2+^ or Sn^2+^), and X is a halide anion (I^−^, Br^−^, Cl^−^). They exhibit tunable bandgaps, high absorption coefficients, long carrier diffusion lengths, and intrinsic defect tolerance [16, 17, 18, 19, 20]. Importantly, they also behave as ionic semiconductors, meaning that both electronic carriers and mobile ionic species (such as halide ions and vacancies) contribute to charge transport [21, 22, 23]. This ionic nature is directly responsible for their memristive behavior, as it enables the dynamic modulation of conductivity through ion migration under an external stimulus [6, 22, 24].

While MHP‐based memristors have demonstrated promising performance in terms of retention time, LRS/HRS ratio, and multilevel switching [25, 26], the fundamental mechanisms driving their resistive switching behavior remain insufficiently understood. This is evidenced by the numerous proposed mechanisms across different devices, often without clear experimental validation [27, 28]. Unlike in conventional oxide‐based RRAMs, where switching mechanisms can often be more distinctly classified [29], MHPs present a more complex scenario due to their soft lattice, low defect formation energies, and simultaneous participation of both vacancies and anionic species in charge transport [30].

The RS behavior in MHP‐based memristors is usually attributed to two mechanisms capable of modulating the device conductivity: electrochemical metallization (ECM) and valence change mechanism (VCM). ECM involves the migration of metal cations (e.g., Ag^+^ or Cu^+^) from an active electrode (e.g., Ag, Cu) into the MHP layer. These species can induce the resistance switch by altering the energetic alignment at the interfaces [31], or by forming metallic (e.g., Ag(0) or Cu(0)) conductive filaments under bias [32], as it is usually reported in non‐MHP‐based memristors [33]. In contrast, VCM is driven by the migration of perovskite halide anions or their vacancies [34]. It is important to emphasize that here we associate the ECM behavior with an active‐metal‐driven mechanism and the VCM with a perovskite‐driven mechanism, without entering into discussions regarding filament formation or interfacial species accumulation.

Distinguishing between ECM and VCM in practice is nontrivial. The interplay between ECM and VCM mechanisms is often complex, particularly in devices where both metal cation migration and halide vacancy dynamics are active [28, 35]. Although replacing the top electrode with an inert metal and observing the absence of switching has been proposed to isolate ECM contributions [36], the lack of systematic reporting in the literature, such as the omission of critical parameters like the bias polarity used during operation, hinders a clearer understanding of the underlying mechanism.

Consequently, while ECM and VCM provide a useful conceptual framework, the specific switching process in MHP memristors is often not clearly identified, and a direct comparison between the performance of both mechanisms is still missing. A deeper experimental understanding is urgently needed to elucidate the fundamental properties and performance of each mechanism. Gaining a clear mechanistic understanding is crucial for improving memristor performance and for guiding the design of reliable perovskite‐based memory and neuromorphic systems.

In this work, we provide the first direct evidence that ECM and VCM switching mechanisms can coexist within the same MHP memristor and can be selectively activated by merely reversing the electroforming voltage polarity, without altering the device structure or the composition of the active layer. This strategy allows a controlled evaluation of both mechanisms under identical conditions. Additionally, replacing the active electrode with two inert contact electrodes enables the isolation of the VCM mechanism, confirming the critical role of active metals in ECM‐driven switching.

By analyzing key performance parameters such as LRS/HRS ratio, endurance, switching voltage, and retention time, we identify which mechanism delivers superior performance for memristive applications.

## 2 Results and Discussion

The standard device architecture employed in this study (Figure S1a) comprises C_60_/BCP and PEDOT:PSS as electron and hole transport layers, respectively, enabling efficient charge transport across the sandwiched MHP layer. Ag was selected as the top electrode. Figure S1b presents a cross‐sectional view of the complete device stack. The active layer, FA_0.9_Cs_0.1_PbI_3_, exhibits a uniform thickness of approximately 500 nm, with well‐defined crystalline grain domains. The Ag electrode has a thickness of 100 nm, whereas the PEDOT:PSS and C_60_/BCP layers are 10 and 30 nm, respectively. The x‐ray diffraction (XRD) results in Figure S1c confirm the high crystallinity of the FA_0.9_Cs_0.1_PbI_3_ layer, with prominent diffraction peaks at (100) and (200), in agreement with the theoretical pattern of the perovskite structure. In the standard measurement configuration, applying forward voltage, hole injection occurs through the bottom PEDOT:PSS layer, while electrons are injected from the top electrode, see Figure S1a. For this configuration, Ag is selected as the top electrode, serving as an active contact for an ECM, which operates only in the presence of metal species with high electrochemical activity. Under an applied bias, Ag^0^ is oxidized at the anode to Ag^+^ and migrates through the perovskite layer driven by the electric field toward the cathode. Ag^+^ can modulate the conductivity by accumulation at the interface, or its reduction to Ag° can lead to the formation of metallic Ag filaments that percolate the MHP film [32, 37].

The electroforming (EF) process is crucial for activating resistive switching in the MHP layer. Although rarely addressed in MHP‐based memristors, the EF protocol, particularly the choice of initiating bias polarity, is strongly influenced by both the device architecture and the layers composition. For our standard devices, EF is initiated by sweeping the applied voltage in the forward direction from 0 to +2 V (Figure 1a, step 1) and from +2 to 0 V (step 2). The voltage sweep continues with the reverse scan, from 0 to −2 V (step 3). During the negative bias, as the voltage is swept back from −2 to 0 V, a spontaneous increase in device conductance is observed (steps 5 and 6), indicating the successful activation of EF. After completing this single voltage cycle (0 V → +2 V → −2 V → 0 V), the device transitions to a stable memristive behavior, with repeatable *I–V* characteristics (Figure 1b), where one can confirm the onsets of resistive switching: a clear set process (HRS to LRS) at positive bias and a reset process (LRS to HRS) at negative bias. Statistical analysis of the cumulative probability distributions (Figure 1c) reveals an average set voltage of +0.18 V and a reset voltage of −0.30 V. The reset process shows slightly higher variability, with a standard deviation of approximately 0.2 V at a 10% confidence level.

**FIGURE 1:**
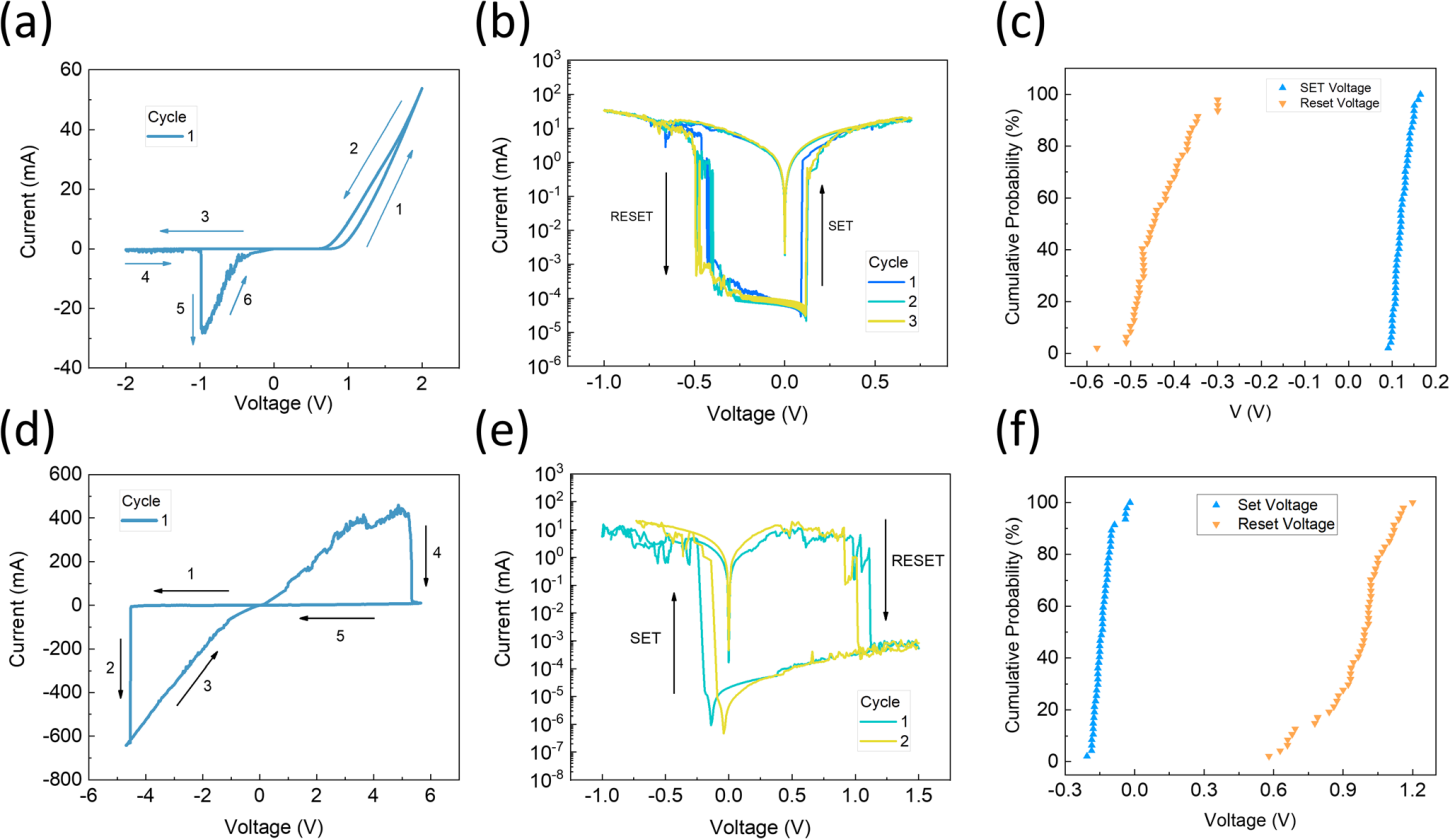
Electroforming process of ITO/PEDOT:PSS/ FA_0.9_Cs_0.1_PbI_3_/C_60_/BCP/Ag with a positive bias 0 V → +2 V → −2 V → 0 V (a), *I–V* characteristics (b), and cumulative probability distributions of set and reset voltages (c) for the ECM mechanism. Electroforming process with a negative bias 0 V → −4.3 V → +5 V → 0 V (d), *I–V* characteristics (e), and cumulative probability distributions of set and reset voltages (f) for the VCM mechanism.

Each step of this EF can be ascribed to the processes of an ECM. Starting from 0 V, the device is initially in an inactive HRS, exhibiting negligible current. At an activation voltage, around +1.1 V (step 1), a sharp current increase marks the transition to LRS, following an ECM: Ag at the top electrode is oxidized to Ag^+^, migrates through the MHP layer, and can accumulate at the interface or be reduced to Ag^0^ to form a metallic filament that switches the device to a LRS. Upon reducing the voltage from +2 V (step 2), the Ag‐induced conductive state becomes unstable and partially or completely relaxes, thereby restoring HRS and enabling reversible resistive switching. The Ag^+^ species generated remain partially trapped within the perovskite matrix, maintaining the device in the HRS over steps 3 and 4.

The importance of the EF characteristics becomes evident when the voltage parameters are modified while maintaining exactly the same device architecture. Specifically, the final device behavior changes if the process starts with the application of a negative bias, in contrast to the previous case shown in Figure 1a. When EF begins with a negative voltage (Figure 1d, step 1), the device conductance increases abruptly at −4.3 V (Figure 1d, step 2), signaling the activation of EF. Step 3 corresponds to the low‐resistance state (LRS), whereas in step 4 the current decreases at +5 V, indicating a transition to the high‐resistance state (HRS). After completing this single voltage cycle (0 V → −4.3 V → +5 V → 0 V), the device establishes stable memristive behavior. Interestingly, the subsequent reproducible *I–V* measurements (Figure 1e) confirm a stable onset of resistive switching. However, in the configuration reached with this EF, the set process occurs at negative voltages, while the reset process occurs at positive voltages. This behavior is the opposite of that observed in Figure 1b, which we previously associated with the ECM mechanism. The statistical analysis of the cumulative probability distributions (Figure 1f) reveals low dispersion in the set voltage, with an average value of −0.15 V. In contrast, the reset process exhibits slightly higher variability, with an average value of +1.1 V and a standard deviation of approximately 0.1 V at a probability level of 70%. The EF mechanism associated with these observations is attributed to a VCM process, as will be discussed in detail below.

These results indicate that the EF protocol defines two distinct switching regimes depending on the polarity of the initial bias. When EF is performed with a 0 V → +2 V → −2 V → 0 V *I–V* sequence, the device exhibits switching consistent with an ECM mechanism. In contrast, when EF begins with negative bias (0 V → −4.3 V → +5 V → 0 V), we observe an inversion in both the polarity and magnitude of the set and reset voltages that is attributed to a VCM mechanism.

From a macroscopic point of view, under a positive EF bias, the applied potential surpasses the standard redox potential of silver (Ag⇌Ag^+^ + e^−^, *E* ^0^ =  0.7996 V), inducing the oxidation of Ag to mobile Ag^+^ cations. These cations can migrate toward the counter electrode and trigger the switch by accumulation at the interface, where they modulate the local energy levels and band alignment, thereby driving the observed conductance change [31] (ECM regime, Figure S2). As an alternative switching mechanism often discussed in the literature, if a sufficient concentration gradient and local field are sustained, the Ag+ cations can nucleate a conductive filament [32].

Conversely, a negative EF voltage generates an opposite electric field that completely suppresses Ag+ cations injection. In this scenario, the resistance change is exclusively governed by the migration of native species within the perovskite material (such as halide ions and vacancies), resulting in a VCM behavior. The electric field forces these mobile ionic species to redistribute and accumulate at the device interfaces or form vacancy‐based filaments. Such VCM process aligns with our previous impedance spectroscopy studies on inert‐electrode devices [4], which explicitly confirmed the diffusion kinetics of these native ionic species during the EF process.

To provide direct physical and chemical evidence of the Ag‐driven ECM switching mechanism, cross‐sectional transmission electron microscopy (TEM) and energy‐dispersive x‐ray spectroscopy (EDS) mapping were performed on electrically activated devices under both ECM and VCM regimes. As depicted in Figure S3a, the TEM image presents the cross‐section of the full device structure, displaying a well‐crystallized active layer with a thickness of approximately 500 nm. After the ECM regime activation, the corresponding silver elemental mapping (Figure S3b, represented by yellow dots) reveals a pronounced accumulation of Ag at both electrode interfaces following activation. This interfacial Ag accumulation aligns closely with recent work [31] that attributed the conductance modulation to the tuning of interfacial energy levels driven by local silver species accumulation. In contrast, for the device operated under the VCM regime, the TEM image (Figure S3c) and Ag EDS mapping with blue dots (Figure S3d) show no silver migration or accumulation, confirming that the VCM process operates independently of active‐metal electromigration and providing robust, direct support for the coexistence of these distinct mechanisms.

After confirming the presence of both mechanisms, ECM and VCM (linked to Ag and perovskite species, respectively), it is crucial to understand how they coexist and dynamically transition within the same device. As shown in Figure 2a, the device initially exhibits characteristic VCM behavior over two consecutive cycles, with the SET process occurring at negative bias and RESET at positive bias. When subjected to a higher negative voltage sweep (Figure 2b), the device first undergoes VCM set voltage until a distinct current transition is triggered around −1.5 V. Following this transition, the subsequent sweep reveals a shift in the set voltage to +0.18 V, signaling the onset of the ECM regime. Figure 2c confirms this stabilized ECM operation over two subsequent cycles, characterized by V_set_ at positive voltages and V_reset_ at negative voltages. In cycle 6 (Figure 2d), the device is exposed to higher positive voltages. After the initial ECM set voltage, reaching approximately +1.4 V causes a sharp current drop, associated with a reverse transition back to the VCM regime. This restoration is verified in the following cycle (Figure 2e), where set voltage recurs at negative bias and reset voltage at positive bias. Finally, Figure 2f provides a direct comparison of the *I*−*V* characteristics from both regimes (green for ECM and orange for VCM), clearly illustrates the polarity inversion, and underscores the coexistence of both switching mechanisms within the same device. These findings highlight the intrinsic complexity of the system and the distinct dynamics of the various ionic species involved in the mechanism. Notably, this represents the first time such coexisting behaviors are reported within a single device during a continuous measurement sequence, opening up new pathways toward a deeper understanding of memristive device physics.

**FIGURE 2:**
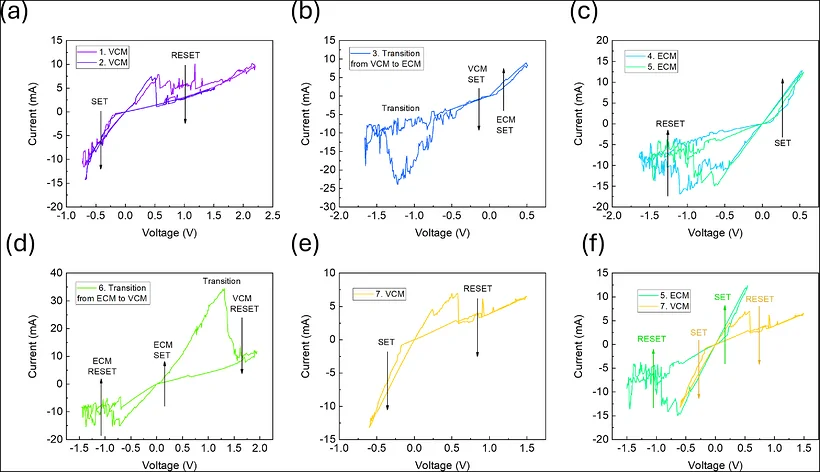
*I–V* curves of the standard configuration ITO/PEDOT:PSS/FA_0.9_Cs_0.1_PbI_3_/ C_60_/BCP/Ag, showing the transition from VCM to ECM and back to VCM polarity in the same device (a–e), and comparison of the curves for both mechanisms (f). The numbers in the legends indicate the cycle number within the continuous measurement sequence.

The role of Ag in the ECM process can be assessed by replacing the active electrode with inert contacts. As a first approach, graphite, a well‐known, low‐reactive semimetal, was directly deposited onto the MHP layer. The device architecture is schematized in Figure S4a. When the EF protocol initiated with a positive‐bias sequence, associated with ECM (0 V → +2 V → −2 V → 0 V *I–V* sequence), the current increase associated with EF occurred at −2 V, while the reset transition appeared at +1.8 V (Figure 3a). After this initial *I–V* cycle, the device stabilizes into a reproducible switching regime with a set voltage of −0.55 V and a reset voltage of +0.8 V, Figure 3b. Remarkably, the polarity of these set/reset transitions corresponds to the behavior previously attributed to the VCM mechanism. When the EF process for this architecture is initiated with a negative‐bias sequence (0 V → −4.3 V → +5 V → 0 V), analogous VCM‐associated curves are observed (see Figure S5).

**FIGURE 3:**
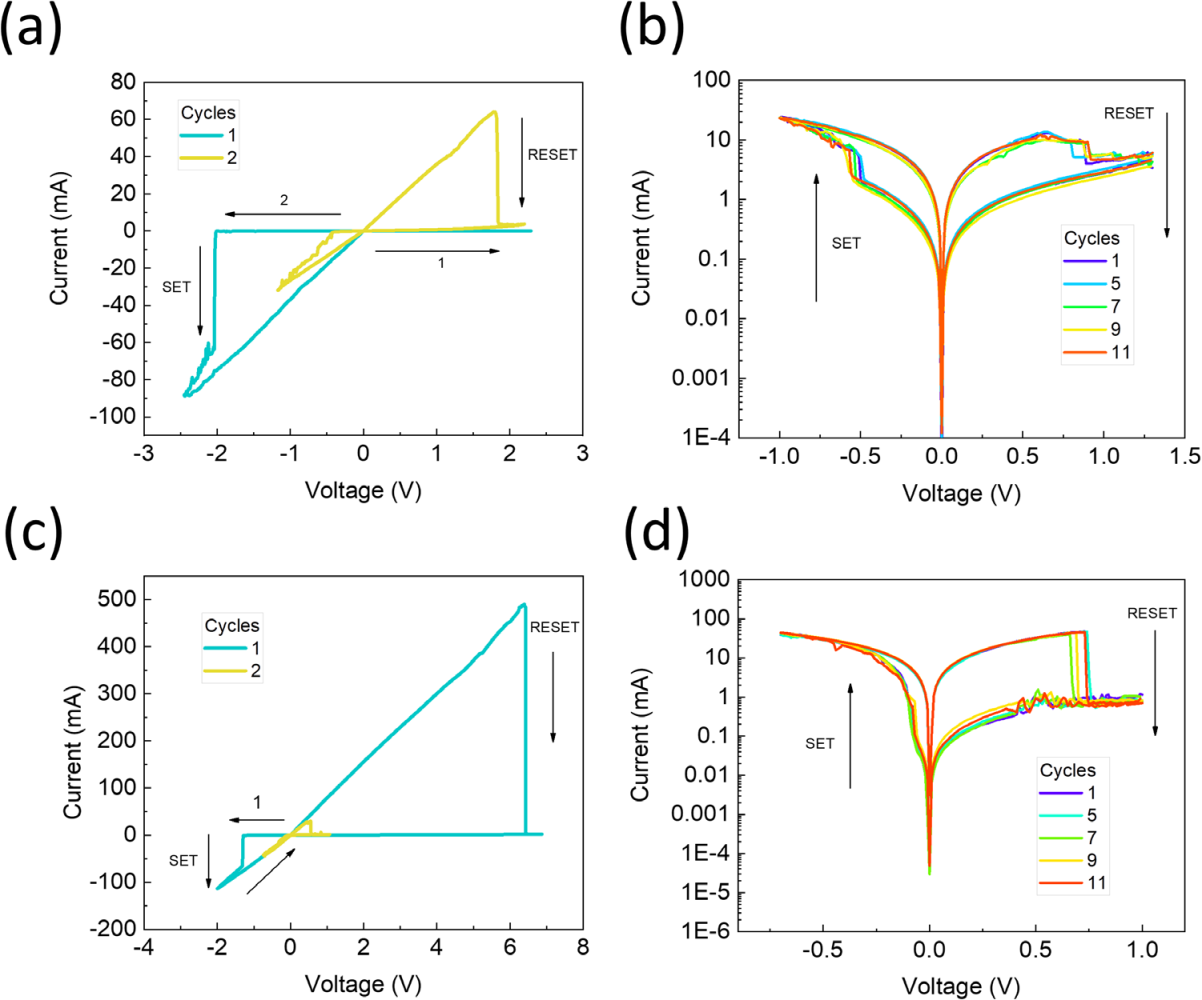
Electroforming process (a) and *I–V* curves (b) for ITO/PEDOT:PSS/ FA_0.9_Cs_0.1_PbI_3_/Graphite device structure. Electroforming process (c) and *I–V* curves (d) for ITO/PEDOT:PSS/ FA_0.9_Cs_0.1_PbI_3_/C_60_/BCP/Au device structure.

To further relate Ag reactivity to the ECM mechanism, we used Au as an inert electrode (see Figure S4b). Au has been proven to migrate, to some extent, across the device, yet its nature is much less reactive than Ag. When the EF protocol was initiated with a positive‐bias sequence (0 V → +2 V → −2 V → 0 V), no resistive switching was observed (Figure S6). However, when the EF process was initiated with a negative bias sequence (Figure 3c, step 1), a conductivity increase occurred at −1.3 V (step 2), followed by a transition to the HRS at +6.5 V. After this *I–V* cycle, the device stabilized into a reproducible resistive switching regime (Figure 3d), with an average set voltage of −0.1 V and a reset voltage of +0.75 V. The absence of switching under a positive‐bias sequence, combined with the stable polarity‐inverted behavior observed under negative‐bias EF, excludes ECM contributions and reinforces that the mechanism governing resistive switching in this configuration is VCM.

In summary, replacing the reactive Ag electrode with inert electrodes (either graphite or Au) excludes an electrochemical metallization mechanism. Under these conditions, resistive switching must be governed by the intrinsic mobile ionic defects of the MHP. These ions, primarily halides and their vacancies, can either migrate to form conductive filaments within the bulk or accumulate at the interfaces [35], thereby modulating the Fermi level alignment and altering the device conductivity. The critical role of MHP ionic migration in resistive switching is widely acknowledged in the community; for example, we recently demonstrated the critical role of mobile ions by monitoring the electroforming process in situ using impedance spectroscopy [4] and their combination to form multistate memristors [38]. In this work, rather than separating filamentary conduction from interfacial ion accumulation, we aim to establish a reliable EF protocol that selectively enables ECM‐ or VCM‐driven switching on demand, depending on the device contact. Although the distinction between ECM and VCM mechanisms is widely discussed in the literature, the polarity sequence applied during electroforming is rarely specified. Our results demonstrate that the initial EF polarity is a decisive parameter that determines the dominant switching mechanism: a positive initial bias preferentially activates ECM behavior, whereas a negative initial bias favors VCM behavior.

The following questions concern the performance of devices with analogous structures operating via VCM and ECM mechanisms, evaluating parameters such as switching voltages, endurance, retention, and variability, and identifying the device architectures that favor one process or the other. The device with the structure ITO/PEDOT:PSS/FA_0.9_Cs_0.1_PbI_3_/C_60_/BCP/Ag, which demonstrates both mechanisms, allows for the first time such a direct comparison. Figure 4a shows the retention characteristics of the ECM mechanism, demonstrating stability over 4000 s for both resistance states. A slight decay in conductance is observed in both HRS and LRS, yet the device maintains an LRS/HRS ratio of ∼ 10^4^ throughout the measurement. The read voltage was set at 20 mV. The endurance test, presented in Figure 4b, confirms reproducible switching over 10^4^ cycles, placing the performance among the best reported in the literature. During cycling, the HRS remains stable, while the LRS exhibits minor instabilities. The set and reset voltages are +0.7 and −1.0 V, respectively, with a read voltage of 20 mV.

**FIGURE 4:**
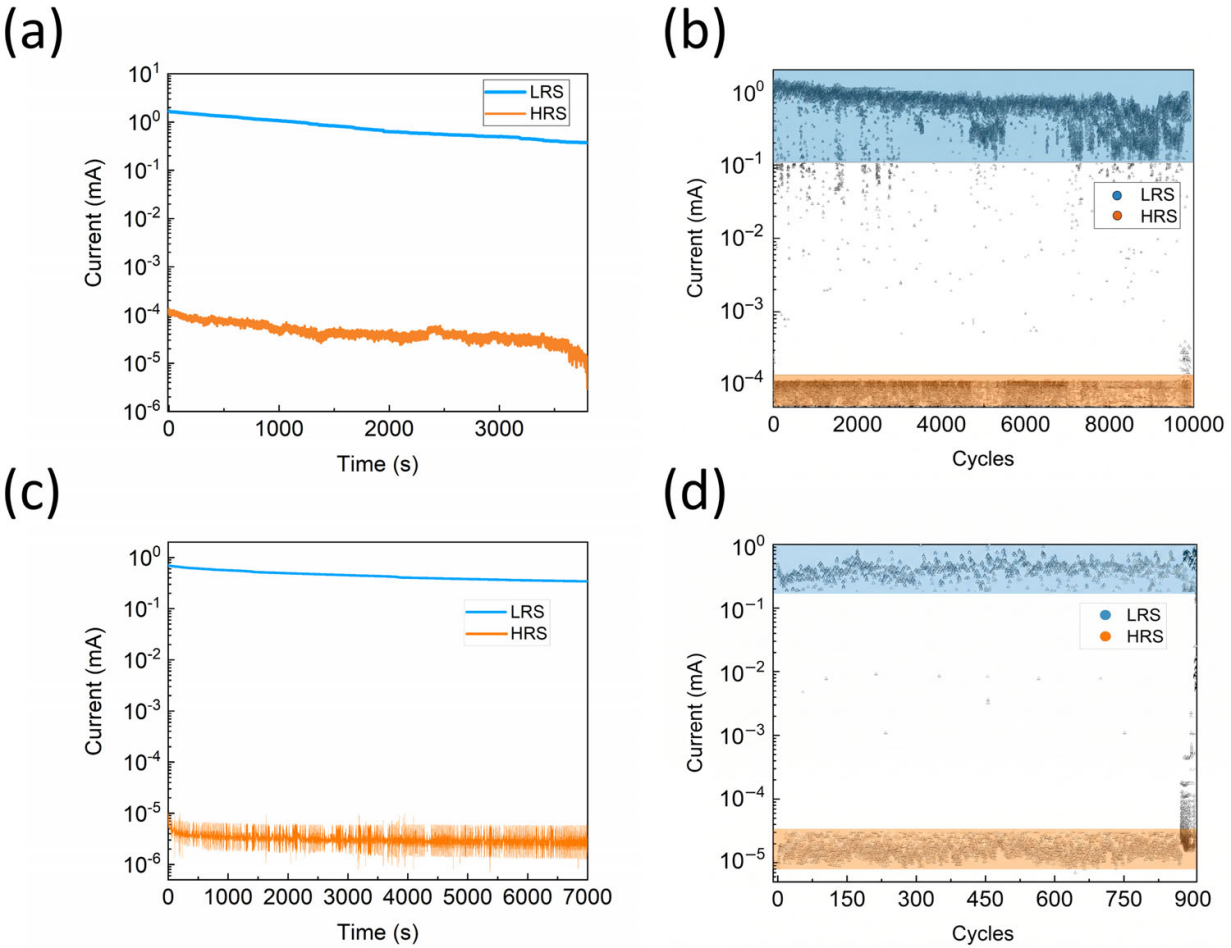
Retention test (a) and endurance test (b) for the standard device operating via the ECM mechanism. Retention test (c) and endurance test (d) for the same standard device configuration operating via the VCM mechanism.

Figure 4c shows the retention characteristics of the VCM mechanism, demonstrating remarkable stability over 7000 s for both states. No appreciable decay in conductance is observed, and the device maintains an LRS/HRS ratio of ∼ 10^5^ throughout the measurement. The read voltage was set at 20 mV. The endurance test, presented in Figure 4d, confirms reproducible switching over 900 cycles, with no significant dispersion in either state. The set and reset voltages are −3.0 and +0.7 V, respectively, with a read voltage of 20 mV.

For both operating mechanisms, the read voltage of 0.02 V was consistently employed during all retention and endurance evaluations. Retention behavior was evaluated by first setting the devices into their LRS or HRS using standard mechanism‐specific I–V sweeps, followed by continuous state readout at a constant bias of 0.02 V over time. Similarly, endurance characteristics were assessed under pulsed operation by probing the resistance states at 0.02 V as the reading voltage, complete details regarding the pulse profile, timing, and sequence parameters are provided in Figure S7.

A direct comparison between the two mechanisms highlights their distinct performance profiles. ECM‐driven switching exhibits excellent endurance, sustaining over 10^4^ cycles, but shows moderate retention (4000 s) and a resistance contrast of ∼ 10^4^. The switching voltages are relatively low, with a set voltage of +0.7 V and a reset voltage of −1.0 V. In contrast, VCM‐driven switching provides superior retention stability (7000 s) and a higher LRS/HRS ratio (∼ 10^5^), although the cycling endurance is limited to ∼ 900 cycles. The required switching voltages are higher in magnitude, with a set voltage of −3.0 V and a reset voltage of +0.7 V. These results reveal a clear trade‐off: ECM offers higher operational robustness, lower operating voltages, and superior endurance, while VCM delivers enhanced resistance contrast and long‐term data retention at the expense of higher switching voltages and reduced cycling stability.

To put these results into perspective, we have included a benchmarking table (Table S1) comparing our performance metrics with the current state of the art in halide perovskite memristors. Notably, this comparison also outlines the different switching mechanisms proposed across the literature, highlighting how our work unifies these distinct operational modes within a single device structure while demonstrating highly competitive endurance, retention, and resistance contrast.

Interestingly, the comparison between the *I–V* curves of the three device architectures that exhibit VCM‐dominated behavior (Figure S8) offers valuable insights. First, comparing the standard architecture (blue line) with the one in which Ag is replaced by Au (yellow line), we observe that the set and reset voltages are reduced in the latter case. However, this improvement comes at the expense of a drastic decrease in the LRS/HRS ratio, which drops by nearly four orders of magnitude. These results highlight that in VCM‐mediated switching, not only the intrinsic mobile ions of the MHP play a critical role in resistive behavior, but also the choice of electrode material can enhance or diminish key properties such as switching voltage and resistance contrast. When examining the device with graphite contact and without the electron transport layer (gray line), the switching behavior becomes more analog‐like, while the LRS/HRS ratio decreases compared to the other two architectures, as the absence of the electron transport layer likely enhances interfacial charge trapping, promoting continuous conductance changes. This transition toward gradual resistance modulation, characteristic of analog switching, at the expense of a reduced on/off window, may affect the device's suitability for digital memory applications.

Remarkably, while the omission of the ETL drastically reduces the ON/OFF window, the fingerprints of the expected VCM‐driven mechanism are evident.

## 3 Conclusions

We have demonstrated a direct comparison of ECM‐ and VCM‐driven switching within the same MHP memristor device with ITO/PEDOT:PSS and C_60_/BCP/Ag as contacts by controlling the polarity of the electroforming protocol. ECM yields low switching voltages (+0.7/−1.0 V) and excellent endurance (> 10^4^ cycles), while VCM provides superior retention (7000 s) and higher resistance contrast (∼ 10^5^), albeit with higher operating voltages (−3.0/+0.7 V) and limited endurance (∼ 900 cycles). Replacing Ag with inert electrodes unambiguously excluded ECM, confirming that intrinsic ionic migration governs VCM. Moreover, electrode composition and charge‐selective layers were found to modulate key parameters such as switching polarity, LRS/HRS ratio, and analog vs. digital behavior in the VCM‐driven mechanism.

These results establish a clear framework for distinguishing between ECM and VCM mechanisms and offer rational design guidelines for tailoring perovskite memristors. By clarifying how switching polarity, electrode activity, and device architecture influence each mechanism, this work enables the systematic optimization of perovskite memristor performance and supports the maturation of a technology that is still in its early stages of development.

## 4 Experimental

### 4.1 Materials

Lead iodide (PbI_2_) and cesium iodide (CsI) were purchased from TCI. Formamidinium iodide (HC(NH_2_)_2_I) was purchased from GreatCell. Poly(3,4‐ethylenedioxythiophene) polystyrene sulfonate (PEDOT:PSS) was purchased from Ossila. N,N‐dimethylformamide (DMF) and Dimethyl sulfoxide (DMSO) were purchased from Thermo Scientific. Chlorobenzene was purchased from VWR. C_60_ fullerene and BCP (bathocuproine) were purchased from TCI. Graphite solution was purchased from RS. All the reagents were directly used without any further purification.

### 4.2 Device Fabrication

The detailed fabrication procedure of the planar ITO/PEDOT:PSS/ FA_0.9_Cs_0.1_PbI_3_/C_60_/BCP/Ag device structure is described below. A 1.2 m perovskite precursor solution was prepared by dissolving 1.08 m of formamidinium iodide (FAI), 0.12 M of cesium iodide (CsI), and 1.2 M of lead iodide (PbI_2_) in 1 mL of a mixed solvent of N,N‐dimethylformamide (DMF) and dimethyl sulfoxide (DMSO) in a 4:1 volume ratio. The solution was stirred overnight at room temperature to ensure complete dissolution and homogeneous mixing of all precursors.

Glass/ITO substrates were sequentially cleaned in an ultrasonic bath using detergent, acetone, deionized water, and absolute ethanol, each for 10 min. The cleaned substrates were then dried under a strong air flow and subsequently subjected to UV–ozone treatment for 20 min to remove residual organic contaminants.

Subsequently, PEDOT:PSS was deposited on the clean substrates through a 0.45 µm PTFE syringe filter, spinning at 5,000 rpm for 30 s, and annealed at 130 °C for 30 min in ambient conditions, after PEDOT:PSS deposition, the substrates were transferred into a N_2_‐filled glovebox for the perovskite deposition. Afterward, 50 µL of the perovskite precursor solution was deposited on top of the clean PEDOT:PSS substrate and was spin coated at 1,000 rpm for 10 s and 6000 rpm for 25 s. After 15 s of the second step, 300 µL of chlorobenzene (CB) was added. The perovskite was annealed at 100°C for 10 min. Finally, the electron transport material (ETM) C_60_ (30 nm), BCP (6 nm), and the electrode, Ag (100 nm), were thermally evaporated under a pressure less than 10^−6^ mbar.

For the ITO/PEDOT:PSS/FA_0.9_Cs_0.1_PbI_3_/C_60_/BCP/Au device structure, the fabrication process was identical to that of the ITO/PEDOT:PSS/FA_0.9_Cs_0.1_PbI_3_/C_60_/BCP/Ag device, except that 100 nm of gold was thermally evaporated instead of silver in the final step.

For the ITO/PEDOT:PSS/FA_0.9_Cs_0.1_PbI_3_/Graphite device structure, after perovskite deposition, the graphite electrode was added on the perovskite film by deposing 10 µL of the graphite solution with a micropipette. The solution evaporates in ≈60 s after deposition, forming a solid graphite layer that acts as an electrode.

### 4.3 Characterization

The crystalline structure was assessed by XRD collected on a Bruker D8 Advanced X‐ray diffractometer with Cu Ka radiation (l = 1.5418 Å) and at a scan rate of 5° min^−1^ for 2*𝜃* angles ranging from 12º to 40º. Scanning electron microscope (SEM) images were captured by an S‐4800 instrument from HITACHI (Tokyo, Japan) operating at 2 kV. *I–V* curves and voltage pulses characterization were measured using a Gamry Interface 1010E. The STEM measurements were performed using Hitachi H‐800 STEM (200 kV) equipped with a Röntec microanalysis system, a Gatan 780 TV camera, and 1392 × 1040 resolution digitization with diffraction pattern processing software.

## Author Contributions

**Ismael Fernandez‐Guillen**: conceptualization, formal analysis, writing – original draft, investigation, methodology. **Omar E. Solis**: methodology, investigation, writing – review and editing. **Pablo F. Betancur**: investigation, methodology, writing – review and editing. **Rafael Abargues**: visualization, writing – review and editing, funding acquisition. **Pablo P. Boix**: data curation, conceptualization, supervision, validation, funding acquisition, writing – review and editing.

## Conflicts of Interest

The authors declare no conflicts of interest.

## Supporting information




**Supporting File**: advs78211‐sup‐0001‐SuppMat.docx.

## Data Availability

The data that support the findings of this study are available from the corresponding author upon reasonable request.
