# Supplementary material for: Polarity‐Controlled Coexistence of Electrochemical Metallization and Valence Change Mechanisms in a Unified Metal Halide Perovskite Memristor
Source: Adv Sci (Weinh). 2026 Oct 26:e78211. Online ahead of print. doi: 10.1002/advs.78211 (PMC13697020; doi:10.1002/advs.78211)
Supplement: Supplementary file 1 [file ADVS-9999-e78211-s001.docx]

*Supporting information*

**Polarity-Controlled Coexistence of Electrochemical Metallization and Valence Change Mechanisms in a Unified Metal Halide Perovskite Memristor**

Ismael Fernandez-Guillen, Omar E. Solis, Pablo F. Betancur, Rafael Abargues, Pablo P. Boix.

**Figure S1.** Standard device structure scheme (a), SEM cross-section of the standard device (b), and XRD pattern of the complete device (c).

**Figure S2.** Schematic illustration of silver oxidation and migration formation during the electroforming process in the ECM mechanism.

**Figure S3.** (a) Cross-sectional TEM image of full device structure. (b) Corresponding Ag EDS mapping (yellow dots) for the ECM regime. (c) Cross-sectional TEM image of the device operated under the VCM regime. (d) Corresponding Ag EDS mapping (blue dots) for the VCM regime.

**Figure S4.** Devices architectures with low reactive electrodes, (a) ITO/PEDOT:PSS/FA_0.9_Cs_0.1_PbI_3_/Graphite, and (b) ITO/PEDOT:PSS/ FA_0.9_Cs_0.1_PbI_3_/C_60_/BCP/Au.

**Figure S5.** EF process with VCM protocol for ITO/PEDOT:PSS/FA_0.9_Cs_0.1_PbI_3_/Graphite device architecture.

**Figure S6.** Unsuccessful electroforming attempt using the ECM protocol in the ITO/PEDOT:PSS/ FA_0.9_Cs_0.1_PbI_3_/C_60_/BCP/Au device architecture.

**Figure S7.** Endurance performance of (a) ECM mechanism with V_set_= 0.7 during0.1 s, V_rest_= -1 V during 5 s, and V_read_= 0.02 V during 0.5 s, and (b) VCM mechanism probed at V_set_= -0.7 during 1 s, V_rest_= -3 V during 1 s, and V_read_= 0.02 V during 0.5 s.

**Figure S8.** I-V curves comparing ITO/PEDOT:PSS/ FA_0.9_Cs_0.1_PbI_3_/C_60_/BCP/Ag (blue line), ITO/PEDOT:PSS/ FA_0.9_Cs_0.1_PbI_3_/C_60_/BCP/Au (yellow line), and ITO/PEDOT:PSS/ FA_0.9_Cs_0.1_PbI_3_/Graphite (black line), devices architectures.

**Table S1.** Benchmarking of key resistive-switching metrics (ON/OFF ratio, Vset, Vreset, endurance, retention, and switching mechanism) for representative perovskite-based memristors versus the devices fabricated in this work.

| **Structure** | **ON/OFF Current Ratio** | **V_set_ [V]** | **V_reset_ [V]** | **Endurance [times]** | **Retention [s]** | **Attributed Mechanism** | **Ref.** |
| --- | --- | --- | --- | --- | --- | --- | --- |
| PET/Pt/CsPbBr_3_/Ag | 10^3^ | -1.32 | +0.70 | 5×10^1^ | 10^5^ | Formation/Rupture of V_Br_ CFs | ^S[1]^ |
| Ag/MAPbI_3_/Ag | 7.5 | ±0.1 | ±0.4 | 7.5×10^1^ | 7.5×10^2^ | Reversible Electrochemical Reactions of Ag and I near the Interface of Ag and MAPbI_3_ under Illumination | ^S[2]^ |
| FTO/CsPbBr_3_/Au | 10^3^ | +1.5 | -1.2 | 10^2^ | 10^3^ | Formation/Rupture of V_Br_ CFs | ^S[3]^ |
| p+-Si/MAPbI_3_/Al | 10^3^ | -3.15 | +2.21 | 2×10^2^ | 10^4^ | Formation/Rupture of V_I_ CFs | ^S[4]^ |
| ITO/MA_3_Bi_2_I_9_/Au | 10^2^ | +1.6 | -0.6 | 3×10^2^ | 10^4^ | Formation/Rupture of V_I_ CFs and Charge Trapping/Detrapping under the Electric Field | ^S[5]^ |
| PET/Multilayer Graphene/MAPbI_3_/Au | 3×10^1^ | +0.68 | -0.5 | 5×10^2^ | 10^4^ | Formation/Rupture of V_I_ CFs | ^S[6]^ |
| Si/SiO_2_/Ti/Pt/MAPbI_3_/Au | 10^3^ | +1 | -1 | 5×10^2^ | 10^5^ | Formation/Rupture of V_I_ CFs | ^S[7]^ |
| Si/SiO_2_/Ti/Pt/CsSnI_3_/PMMA/Ag | 7×10^3^ | +0.13 | -0.08 | 6×10^2^ | 7×10^3^ | Formation/Rupture of Ag CFs | ^S[8]^ |
| Au/MAPbI_3_/Au | 10^8^ | +1.16 | +0.46 | 10^3^ | 10^4^ | Formation/Rupture of V_I_ CFs | ^S[9]^ |
| FTO/CsPbBr_3_/Au | 10^5^ | +0.7 | -0.9 | 10^3^ | 10^4^ | Formation/Rupture of V_Br_ CFs | ^S[10]^ |
| FTO//MAPbI_3-x_Cl_x_/Ag | 5×10^1^ | +1.5 | -0.9 | 3×10^4^ | 6×10^3^ | Formation/Rupture of Ag CFs | ^S[11]^ |
| PET/ITO/PMMA/CsPbBr_3_QDs/PMMA/Ag | 6×10^5^ | +2.6 | -2.7 | 5×10^3^ | 4×10^5^ | Formation/Rupture of Ag and V_Br_ CFs | ^S[12]^ |
| Ag/MAPbCl_3_ NWs/Al | 10^7^ | +3 | -2.5 | 10^6^ | — | Formation/Rupture of Ag CFs | ^S[13]^ |
| This work | ~10^4^ | +0.18 | -0.3 | >10^4^ | 4×10^3^ | ECM, oxidation and accumulation of Ag |  |
| This work | ~10^5^ | -1 | +0.7 | 900 | 7×10^3^ | VCM, intrinsic perovskite ion migration) |  |

**References**

S[1]	P. Cheng, Y. Zhu, J. Shi, H. Wang, Y. Liu, R. Xiong, J. Wei, H. Ma, M. Yin, *Appl. Phys. Lett.* **2019**, *115*, 223505.

S[2]	X. Zhu, W. D. Lu, *ACS Nano* **2018**, *12*, 1242.

S[3]	X. Wang, N. Ali, G. Bi, H. Wu, *Semicond. Sci. Technol.* **2022**, *37*, 065011.

S[4]	D. J. Kim, Y. J. Tak, W. Kim, J. K. Kim, J. H. Kim, H. J. Kim, *Adv. Mater. Interfaces* **2017**, *4*, 1601035.

S[5]	B. Hwang, J.-S. Lee, *Nanoscale* **2018**, *10*, 8578.

S[6]	Y. Huang, L. Tang, C. Wang, H. Fan, Z. Zhao, H. Wu, M. Xu, R. Shen, Y. Yang, J. Bian, *ACS Appl. Electron. Mater.* **2020**, *2*, 3695.

S[7]	B. Hwang, J. Lee, *Adv. Mater.* **2017**, *29*, 1701048.

S[8]	J. S. Han, Q. V. Le, J. Choi, H. Kim, S. G. Kim, K. Hong, C. W. Moon, T. L. Kim, S. Y. Kim, H. W. Jang, *ACS Appl. Mater. Interfaces* **2019**, *11*, 8155.

S[9]	K. Kang, H. Ahn, Y. Song, W. Lee, J. Kim, Y. Kim, D. Yoo, T. Lee, *Adv. Mater.* **2019**, *31*, 1804841.

S[10]	X. Wang, N. Ali, G. Bi, H. Wu, *Semicond. Sci. Technol.* **2022**, *37*, 065011.

S[11]	E. J. Yoo, M. Lyu, J. Yun, C. J. Kang, Y. J. Choi, L. Wang, *Adv. Mater.* **2015**, *27*, 6170.

S[12]	Y. Wang, Z. Lv, Q. Liao, H. Shan, J. Chen, Y. Zhou, L. Zhou, X. Chen, V. A. L. Roy, Z. Wang, Z. Xu, Y. Zeng, S. Han, *Adv. Mater.* **2018**, *30*, 1800327.

S[13]	Y. Zhang, S. Poddar, H. Huang, L. Gu, Q. Zhang, Y. Zhou, S. Yan, S. Zhang, Z. Song, B. Huang, G. Shen, Z. Fan, *Sci. Adv.* **2021**, *7*, eabg3788.
